# DCP-TransUNet: An Approach for Crack Segmentation on Roads

**DOI:** 10.3390/s26031071

**Published:** 2026-02-06

**Authors:** Yunqing Liu, Xu Du, Weiguang Li

**Affiliations:** 1 School of Electronic Information and Engineering, Changchun University of Science of Technology, Changchun 130118, China; mzlyq@cust.edu.cn (Y.L.); 2023200138@mails.cust.edu.cn (X.D.); 2 School of Electronic Information and Engineering, Changchun Guanghua College, Changchun 130118, China

**Keywords:** image segmentation, road cracks, deep learning, TransUNet

## Abstract

For cement pavements on vast road networks, cracking has become one of the principal distresses threatening structural integrity and traffic safety. This study introduces DCP-TransUNet, a model featuring a new hybrid encoder that enhances the continuity of crack extraction under complex conditions through a DSE-CNN module and a CLMA-Transformer block. To further strengthen learning and interpretability for challenging crack imagery, a PPA bottleneck module is designed to capture additional discriminative features. Experimental results indicate strong performance: on the public dataset, DCP-TransUNet achieves mIoU 79.12%, Recall 87.96%, F1 87.06%, and Precision 86.21%; on the private dataset, it attains mIoU 68.83%, Recall 74.42%, F1 77.57%, and Precision 81.67%. Compared with other models, these outcomes demonstrate the method’s accuracy and effectiveness for crack segmentation.

## 1. Introduction

As the most widely used form of transportation infrastructure, roadways are vital to societal operations. Because they are subjected to long-term heavy traffic and freight loads, they are highly susceptible to environmental erosion and loading effects, leading to the gradual emergence of performance problems [1,2,3,4,5]. Among these, cracks are an important indicator of roadway performance degradation; they seriously compromise the integrity of the road structure and, over time, expand further, shortening service life and increasing the risks of traffic accidents and maintenance costs [6,7]. Therefore, how to detect pavement cracks in a timely and accurate manner has become a key problem in road maintenance.

Traditional crack-detection methods have primarily relied on manual patrolling and classical vision-based image processing techniques, such as thresholding [8], edge detection [9], and clustering [10]. Li et al. [11] proposed a crack-detection approach based on dual-threshold segmentation, employing the Otsu thresholding algorithm together with an adaptive iterative thresholding scheme to remove pavement markings and perform image segmentation. Zhao et al. [12] proposed an improved Canny edge detection algorithm by combining the Mallat wavelet transform with an edge filter, focusing on eliminating noise interference and detecting edge cracks in pavement images. Zalama et al. [13] proposed using Gabor filters to extract visual features and the AdaBoost algorithm to reselect and combine classifiers to detect pavement cracks. Shi et al. [14] proposed a crack-detection framework based on a random-forest architecture and designed a novel crack descriptor to discriminate cracks from noise. Zhou et al. [15] detected crack seed points via grid-cell analysis and then connected crack points by constructing a Euclidean minimum spanning tree. Subsequently, unnecessary noise was removed to enable pavement-crack detection.

These methods face low segmentation accuracy under complex road conditions, such as shadows, occlusions, and tiny cracks, are not applicable to a wide range of scenarios, and struggle to meet the timeliness and accuracy requirements of modern road maintenance.

With the rapid progress of deep learning, convolutional neural network (CNN)–based approaches to crack detection and segmentation have emerged as a central research focus [16]. By capitalizing on neural networks’ strengths in feature extraction and segmentation accuracy [17,18], the reliability of roadway crack delineation has been improved, thereby providing a stronger scientific basis for maintenance planning [19,20,21]. DeepLabV3+ was enhanced by Li et al. [22] using an adaptive probabilistic sampling strategy and an external attention mechanism to segment pavement defects. An encoder–decoder architecture that integrates the Swin Transformer with UPerNet was introduced by Guo et al. [23] for precise crack detection. Efficient pavement-crack segmentation was achieved by Blessing Agyei Kyem et al. [24] through the incorporation of a Region-Focused Enhancement Module and a Context-Aware Global Module within an encoder–decoder framework. Ji et al. [25] fused a convolutional encoder with self-attention and proposed a Transformer-based TransUNet model for pavement crack detection. Qi et al. [26] adopted GhostNet as the baseline model and proposed GMDNet by incorporating dynamic convolution and a multi-scale convolutional attention aggregation module, enabling accurate and efficient segmentation of irregular pavement cracks. Zhang et al. [27] proposed MixCrackNet, a pavement-crack segmentation model that integrates deformable convolution, a weighted loss function, and an efficient multi-scale attention module to identify pavement cracks.

Despite substantial gains in automation and accuracy, existing crack detection and segmentation models still face several challenges. The slender morphology of cracks can prevent networks from covering sufficiently long extents to preserve continuity. Even when continuous crack patterns are present, portions may be obscured by specific convolution kernels, leading to incomplete extraction. Moreover, the diversity of roadway environments introduces confounding factors—such as shadows cast by roadside trees—that further hinder reliable crack identification.

To address these issues, a DCP-TransUNet model is proposed. A hybrid encoder composed of DSE-CNN and CLMA-Transformer is employed to generate high–signal-to-noise, multi-scale semantic representations. In addition, a PPA-bm module is introduced to achieve pixel-level alignment and dual-scale patch awareness, providing stable support for fine-grained reconstruction in the decoder and thereby markedly improving the continuity and accuracy of crack segmentation. The principal contributions are summarized as follows:

1. A rural cement-pavement crack dataset is constructed that explicitly includes confounders such as shadows and specular reflections. The dataset contains 2720 images of irregular cracks against complex backgrounds and is used to train the proposed model for pavement-crack segmentation.
2. To address the blurring and low recognizability of cracks caused by numerous confounding factors in in situ roadway images, a DSE-CNN module is introduced. Within each Pre-Activation residual bottleneck, dual-channel recalibration driven by global average pooling and max pooling is performed to amplify informative responses and suppress irrelevant activations, thereby providing a more stable encoded input.
3. Given the elongated, ribbon-like geometry and pronounced directionality of pavement targets, conventional multi-head attention lacks explicit orientation modeling. Accordingly, a CLMA-Transformer Block is proposed, in which cross-stripe attention establishes directional dependencies and memory tokens aggregate global context, better accommodating the slender morphology of cracks and improving detection performance.
4. To remedy the limitation of conventional bridges that transmit only high-level semantics—leading to sub-stride cracks being diluted before upsampling and poorly aligned with low-level skip features—a PPA bottleneck module (PPA-bm) is incorporated. This module refines and supplements boundary gradients via convolution, reinforces fragment continuity through dual-scale patch awareness, and fuses features with channel-wise and spatial-wise gated weighting, thereby enhancing deep feature extraction, improving segmentation accuracy, and reducing redundancy.

## 2. Materials and Methods

**Figure 1:**
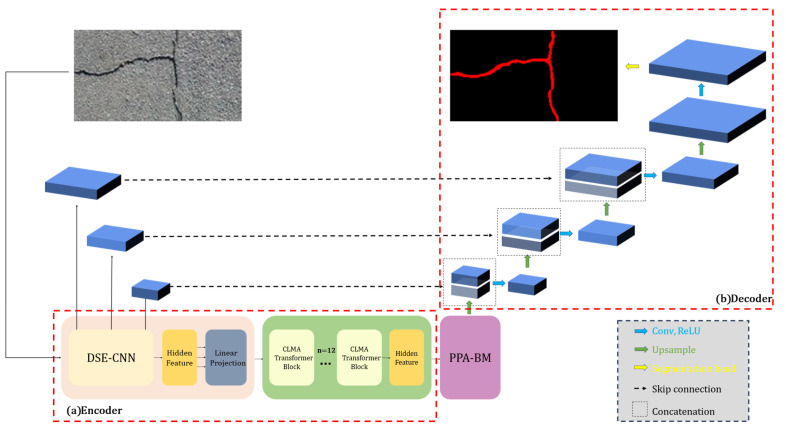
Overall architecture of the proposed DCP-TransUNet model: (**a**) encoder stage; (**b**) decoder stage.

A hybrid DCP-TransUNet network is proposed on the basis of the TransUNet architecture (Figure 1). A new encoder is designed in which DSE-CNN performs dual-channel recalibration—driven by global average pooling and global max pooling—within each Pre-Activation residual bottleneck. Channel responses are twice selectively screened and rescaled to amplify target-relevant information while suppressing background redundancy and noise, thereby providing a more stable and discriminative input for subsequent feature representation. In addition, a CLMA-Transformer Layer is introduced to establish directional dependencies via horizontal–vertical cross-stripe attention, while a small set of memory tokens aggregates cross-window global context. In this way, the orientations and continuity of fine-grained targets are better captured, alleviating the boundary insensitivity and computational redundancy that arise from isotropic modeling over fixed patch grids in conventional multi-head self-attention.

To address the limitation of conventional bridges that transmit only high-level semantics—leading to sub-stride, fine features being diluted during upsampling and poorly aligned with low-level skip connections—a Parallelized Patch-Aware bottleneck is incorporated. This module refines and supplements boundary gradients through convolution, consolidates fragment consistency via dual-scale patch awareness, and performs channel- and spatial-level gated fusion to complete token–pixel alignment and de-redundancy before the decoder. Finally, a U-Net–style four-stage upsampling decoder is employed. The encoder output is first integrated with a 3 × 3 convolution, then progressively fused—via four upsampling stages—with multi-scale skip features from the encoder, and a lightweight SegmentationHead is used to produce the final segmentation.

### 2.1. DSE-CNN

**Figure 2:**
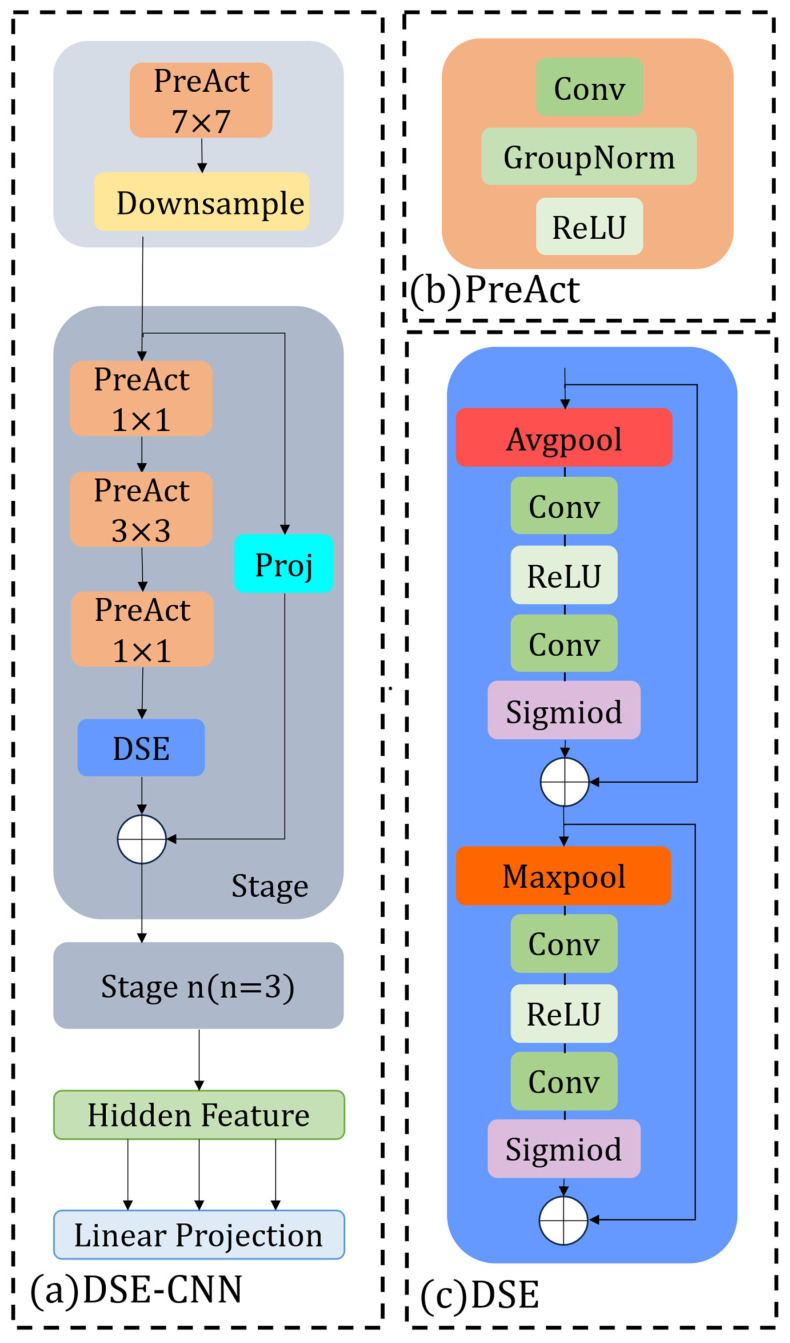
(**a**) DSE-CNN architecture; (**b**) Pre-Activation residual encoder; (**c**) Dual Squeeze–Excitation module.

In pavement-segmentation settings, shadows, specular reflections, and fine textures readily perturb low- and mid-level channel responses, leading to blurred boundaries and breaks in slender structures. To enhance the encoder’s selectivity for discriminative channels, a Dual Squeeze–Excitation (DSE) mechanism is incorporated into the four-stage Pre-Activation residual encoder. Within each bottleneck block, and immediately before the summation of the main branch with the short residual, channel-wise recalibration is applied to the main-branch output, as illustrated in Figure 2.

The Dual Squeeze–Excitation module first extracts two types of global channel statistics, as shown in Equation (1). Let the main-branch convolutional output be $Y\in R^{B\times C\times H\times W}$.


(1) $$\{\begin{array}{c} GAP\left(x\right)=\frac{1}{HW}\sum_{h=1}^{H}\sum_{w=1}^{W}Y_{b,c,h,w}\\ GMP\left(x\right)={max}_{1\leq h\leq H,1\leq w\leq W}Y_{b,c,h,w} \end{array}$$

These statistics are then passed through a lightweight channel MLP implemented with 1×1 convolutions and ReLU/Sigmoid activations to produce two channel-attention maps. Sequential recalibration is applied to Y, yielding the updated output in Equation (2). Where ⊙ denotes channel-wise multiplication with broadcasting over spatial dimensions


(2) $$\{\begin{array}{c} s_{avg}=Sigmoid\left({Conv}_{1\times 1}\left(ReLU\left({Conv}_{1\times 1}\left(GAP\left(x\right)\right)\right)\right)\right)\\ x_{1}=x⊙s_{avg}\\ s_{max}=Sigmoid\left({Conv}_{1\times 1}\left(ReLU\left({Conv}_{1\times 1}\left(GMP\left(x_{1}\right)\right)\right)\right)\right)\\ y=x_{1}⊙s_{max} \end{array}$$

By employing the improved encoder, background responses unrelated to the roadway are effectively suppressed, while discriminative features associated with road boundaries and slender crack structures are enhanced. As a result, boundary drift and structural discontinuities are mitigated, and stable, separable multi-scale representations are provided for subsequent training.

### 2.2. CLMA-Transformer Block

In crack segmentation, targets typically exhibit slender, ribbon-like geometries with pronounced directional consistency along the road’s normal/tangential axes. Meanwhile, shadows, specular reflections, fallen leaves, and vehicle occlusions can introduce local interruptions and topological discontinuities. Attention mechanisms are well suited to establish long-range dependencies among pixels and are therefore crucial for preserving the global connectivity of crack/road centerlines and achieving cross-occlusion alignment. In the conventional TransUNet, multi-head self-attention projects input tokens into (Q,K,V) via multi-head linear mappings, computes scaled dot-product similarities over all token pairs, and aggregates them globally with a Softmax weighting, as shown in Equation (3).


(3) $$MSA\left(Z\right)={Concat}_{h}\left[Softmax\left(\frac{Q_{h}K_{h}^{⊤}}{\sqrt{d}}\right)V_{h}\right]$$

Although MSA can capture certain long-range dependencies for crack segmentation, its use of fixed-size patches to compute global similarities often leads to “windowing” artifacts and blurred boundaries when dealing with slender, elongated cracks. Moreover, single-layer global modeling over the entire image is easily dominated by strong local noise, resulting in pseudo-connectivity and spurious diffusion.

To resolve these issues, a CLMA-Transformer Block is proposed. Within a single attention unit, two components are tightly coupled: a cross-stripe attention with locally enhanced positional encoding, which models directional local geometry along the target trajectory and improves boundary adherence, and a global memory cross-attention, which leverages a small set of memory anchors to achieve cross-window global alignment and repair discontinuities.

**Figure 3:**
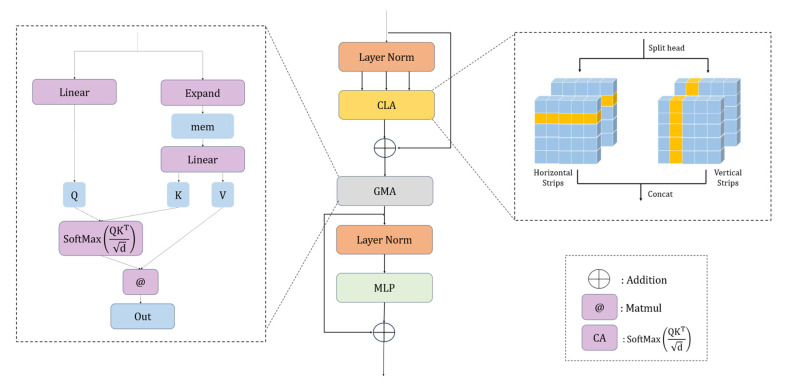
Architecture of the CLMA-Transformer block.

As illustrated in Figure 3, the module adopts a serial, two-sublayer design that couples local cross-stripe attention with global memory cross-attention. First, the input feature Z is layer-normalized and linearly projected to obtain (Q,K,V). These tensors are then evenly partitioned along the channel dimension into horizontal and vertical branches, as expressed in Equation (4).


(4) $$\left[Q_{h},Q_{v}\right]={Split}_{C}\left(Q\right),\left[K_{h},K_{v}\right]={Split}_{C}\left(K\right),\left[V_{h},V_{v}\right]={Split}_{C}\left(V\right)$$

Self-attention is then computed independently within each elongated stripe window so that information is preferentially propagated along the principal axis of the crack. In parallel, a Local-enhanced Positional Encoding is generated by applying depthwise separable convolution to the value branch V; the resulting LePE is added to the attention output. Finally, the outputs from the horizontal and vertical branches are concatenated along the channel dimension and linearly projected to produce the block output, as formulated in Equation (5).


(5) $$\{\begin{array}{c} A_{h}=Softmax\left(\frac{Q_{h}K_{h}^{⊤}}{\sqrt{d}}\right)V_{h}+DWconv\left(V_{h}\right)\\ A_{v}=Softmax\left(\frac{Q_{v}K_{v}^{⊤}}{\sqrt{d}}\right)V_{v}+DWconv\left(V_{v}\right)\\ A_{out}=Proj\left(Concat\left[A_{h},A_{v}\right]\right) \end{array}$$

Subsequently, the stripe-based representation is residually linked with the original input and passed to a global memory retrieval–aggregation stage. A set of Memory Tokens is instantiated by copying learnable parameters along the batch dimension to form Mem, as specified in Equation (6).


(6) $$M={Broadcast}_{B}\left(\theta_{mem}\right)$$

All patch tokens are then used as queries q, while the memory set Mem supplies the keys $K_{mem}$ and values $V_{mem}$. A single scaled dot-product cross-set matching and weighted aggregation is performed, as in Equation (7):


(7) $$\{\begin{array}{c} Q=xW_{q},K_{M}=MW_{k},V_{M}=MW_{v}\\ \hat{Z}=SoftMax\left(\frac{QK^{T}}{\sqrt{d}}\right)V_{M} \end{array}$$

Finally, the result is processed by layer normalization followed by a feed-forward FFN to produce the updated representation, as given in Equation (8).


(8) $$Z^{\prime }=\hat{Z}+FFN\left(LN\left(\hat{Z}\right)\right)$$

In summary, CLMA couples “direction-selective stripe-based local modeling” with “memory token–based global cross-attention” within a single layer. The former, implemented via cross-stripe attention and LePE, strengthens the continuity of slender structures and improves boundary adherence; the latter employs memory anchors to stabilize cross-window dependencies and suppress pseudo-connectivity and spurious diffusion induced by occlusions and specular highlights. The module integrates seamlessly with the TransUNet decoder and exhibits stable training behavior.

### 2.3. Parallelized Patch-Aware Bottleneck

In encoder–decoder architectures, bottleneck blocks play a pivotal bridging role: they must distill and refine high-level semantics on the encoder side while also supplying context that is sufficiently rich and readily projectable into the pixel domain, so that fine cracks and sharp boundaries can be faithfully recovered during upsampling. In the conventional TransUNet, the bottleneck feeds predominantly high-level semantics directly into the decoder; as a result, cracks that are thinner than—or comparable to—the stride are often diluted before upsampling. The decoder then struggles to extract precise boundary details, pixel-level refinements are lacking, post-upsampling boundaries become less sharp, and stable alignment with low-level skip connections is difficult to achieve.

**Figure 4:**
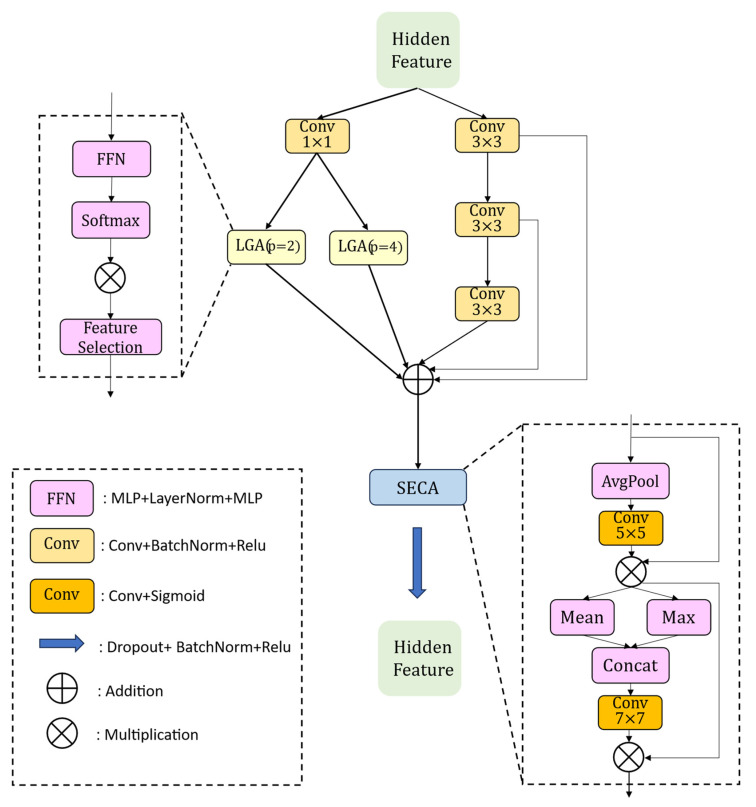
Architecture of the Parallelized Patch-Aware bottleneck.

To remedy these issues, a Parallelized Patch-Aware bottleneck (PPA-bm) is introduced (Figure 4). Without altering the U-shaped skip pathway, the module refines and supplements boundary gradients via convolution, stabilizes fragment consistency and fine-texture continuity through dual-scale patch-aware branches, and employs two-stage channel–spatial gating to accentuate target-relevant components from both the channel and pixel perspectives. A parallel, additive fusion design is adopted to reduce thin-line breakpoints and boundary drift while keeping channel capacity and computational cost under control. The module demonstrates enhanced robustness in challenging scenes with shadows, specular highlights, and local occlusions.

The processing pipeline is as follows. Let the bottleneck input be $x\in R^{B\times C\times H\times W}$. First, a 1×1 convolution followed by normalization produces a pass-through branch $x_{\mathrm{skip}}$, used solely for channel alignment and direct information flow. Next, two patch-aware parallel branches are constructed. Non-overlapping grids with patch sizes P∈{2,4} are applied to $x_{\mathrm{skip}}$ to extract patch sequences. Each patch is first aggregated across channels into a vector, passed through a two-layer MLP and LayerNorm to obtain a response, and then self-gated via a Softmax that suppresses low-confidence components. A second semantic gating is performed using the cosine similarity with a learnable prompt vector, yielding two patch-aware feature maps $x_{\mathrm{lga},2}$ and $x_{\mathrm{lga},4}$.

Meanwhile, to compensate for texture and boundary gradients, the original input x is processed by a serial stack of three lightweight 3×3 convolutional units, producing $c_{1},c_{2},$ and $c_{3}$. Finally, the six streams are summed in the pixel domain to form the updated bottleneck output, as in Equation (9):


(9) $$\{\begin{array}{c} X_{\mathrm{skip}}={\mathrm{Conv}}_{1\times 1}\left(X\right).\\ X_{\mathrm{lga}}^{\left(P=2\right)}=FeatureSelection\left(\mathrm{Softmax}\left(FFN\left(X_{\mathrm{skip}}\right)\right)+FFN\left(X_{\mathrm{skip}}\right)\right)\\ X_{\mathrm{lga}}^{\left(P=4\right)}=FeatureSelection\left(\mathrm{Softmax}\left(FFN\left(X_{\mathrm{skip}}\right)\right)+FFN\left(X_{\mathrm{skip}}\right)\right)\\ X_{1}={\mathrm{Conv}}_{3\times 3}\left(X\right)\\ X_{2}={\mathrm{Conv}}_{3\times 3}\left(X_{1}\right)\\ X_{3}={\mathrm{Conv}}_{3\times 3}\left(X_{2}\right)\\ X_{\Sigma }=X_{\mathrm{skip}}+X_{1}+X_{2}+X_{3}+X_{\mathrm{lga}}^{\left(2\right)}+X_{\mathrm{lga}}^{\left(4\right)} \end{array}$$

Next, two-stage recalibration is applied to $X_{\Sigma }$ in the channel and spatial domains. First, global average pooling compresses each channel u over space, after which an adaptive convolution is performed along the channel axis to mitigate information loss. The result is mapped by a Sigmoid to obtain channel weights, which are then multiplied by the input feature on a per-channel basis. Subsequently, the channel-wise mean and max maps are concatenated and passed through a 7×7 convolution followed by a Sigmoid to yield a pixel-level saliency map for spatial recalibration. Finally, the output is normalized to produce the bottleneck result Y, which is forwarded to the decoder’s subsequent upsampling stages. This procedure is summarized in Equation (10):


(10) $$\{\begin{array}{c} X_{c}=X_{\Sigma }⊙\sigma \left({\mathrm{Conv}}_{1D}\left(\mathrm{GAP}\left(X_{\Sigma }\right)\right)\right)\\ X_{s}=X_{c}⊙\sigma \left({\mathrm{Conv}}_{7\times 7}\left(\left[{\mathrm{Mean}}_{c}\left(X_{c}\right),{\mathrm{Max}}_{c}\left(X_{c}\right)\right]\right)\right) \end{array}$$

### 2.4. Data Sources and Preprocessing

**Figure 5:**
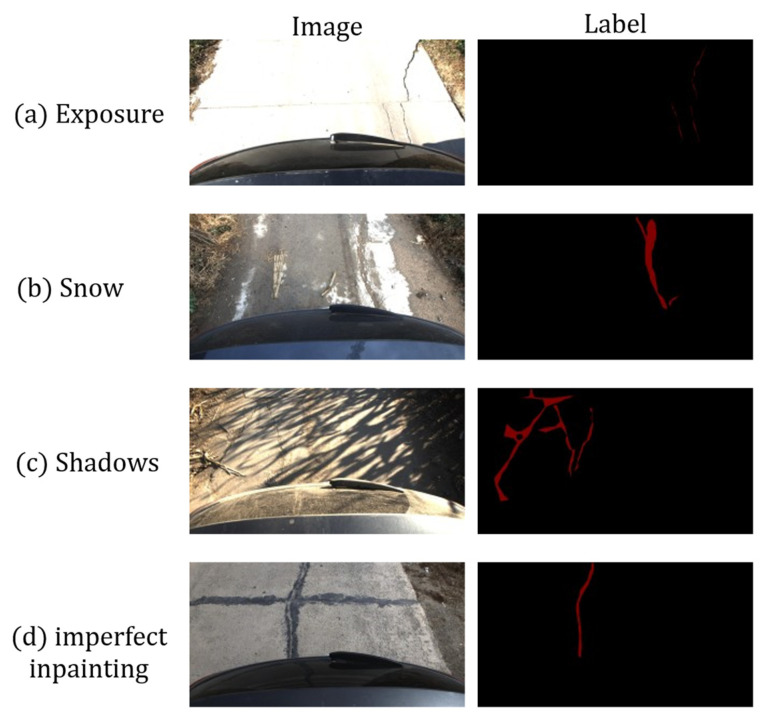
Private Dataset Overview.

Experiments were conducted on the public Crack500 dataset [28] and on a private dataset. Crack500 consists of images captured by a smartphone (approximately 640 × 360 pixels) and their pixel-level annotations. The private dataset was collected by our team in Changchun and its surrounding areas during two daytime acquisition periods. To evaluate the robustness of the model under complex lighting conditions, challenging scenarios such as cast shadows, non-uniform illumination, and snow cover were intentionally included. The image data were acquired using an onboard high-resolution camera, and during acquisition the vehicle traveled at an approximately constant speed of about 20–30 km/h. After preliminary screening and quality control, a total of 2720 representative crack images were retained. For intuitive visualization of the data distribution and typical hard cases, representative samples containing special scenarios such as shadows, non-uniform illumination, and snow cover were selected for visualization, as shown in Figure 5. Subsequently, pixel-level ground-truth masks (crack/background) were generated by three domain experts using the LabelMg annotation tool, where crack pixels were marked in red and background pixels were marked in black. To ensure the consistency and reproducibility of the annotations, a unified pixel-level annotation protocol was established: crack pixels were defined as the visible physical region of pavement cracks, and the boundaries were required to follow the true contours as closely as possible while avoiding artificial outward expansion; for cases involving occlusions such as shadows, reflections, stains, fallen leaves, or snow cover, only the visible parts of cracks were annotated, and no inferential completion was performed for completely invisible regions; non-crack structures, including road lane markings, regular joints, cut joints, and strongly reflective strips, were all treated as background. For quality control, trial annotations and protocol alignment were first carried out on a small subset containing difficult cases such as shadows and intersections, and a consistent list of rules was formed; then, during formal annotation, self-checking and hard-sample marking were performed, and each batch of data was subjected to random spot checks and a complete review of hard samples; for samples with disagreements, the final masks were confirmed after group discussion or adjudication by a senior annotator. All annotation results were subsequently normalized and exported in PNG format at an approximate resolution of 2000 × 1500 pixels for subsequent model training. In all experiments, the dataset was randomly split into training, validation, and test sets at a ratio of 7:1.5:1.5.

## 3. Results and Discussion

### 3.1. Experimental Setup

All experiments were performed on a high-performance Windows-based desktop workstation (self-assembled, Changchun, China) equipped with an NVIDIA GeForce RTX 5090D GPU (Santa Clara, CA, USA). All models were implemented in Python 3.12. To ensure fairness across comparative and ablation studies, identical training settings were used for all models: the maximum number of training epochs was set to $E_{max}=300$, the batch size was fixed at B=4, and all input images were resized to 224×224 prior to being fed into the network. Model parameters were optimized using Adam.

The initial learning rate was set to $1\times 10^{-3}$, which falls within a commonly used range for Adam in image segmentation tasks. A local sensitivity analysis was additionally conducted around this value, and $1\times 10^{-3}$ was selected based on validation performance and the stability of the training curves. It was observed that larger learning rates induced oscillations under certain configurations, whereas smaller learning rates slowed convergence. The batch size was primarily constrained by GPU memory, given the model size and input resolution. Within the feasible memory budget, adjacent batch sizes were also evaluated; however, no consistent and statistically meaningful performance improvements were observed. Therefore, B=4 was used throughout all experiments.

### 3.2. Evaluation Metrics

In this study, precision, recall, mean Intersection over Union (MIoU), and F-score are used as the primary metrics for evaluating model performance. Let K denote the number of classes (here, typically crack/background, so K=2). Pixel-level true positives (TP), false positives (FP), and false negatives (FN) are defined in the standard way.

Mean Intersection over Union (MIoU) is a standard metric in semantic segmentation that quantifies the overlap quality between the predicted mask and the ground-truth mask, as defined in Equation (11).


(11) $$MIoU=\frac{1}{K}\sum_{k=1}^{K}\;\frac{TP_{k}}{TP_{k}+FP_{k}+FN_{k}}$$

Recall measures the proportion of truly positive pixels that are successfully identified, emphasizing completeness of target coverage; it is commonly used to assess a model’s ability to control omissions, as defined in Equation (12).


(12) $${Recall}_{k}=\frac{TP_{k}}{TP_{k}+FN_{k}}$$

Precision measures the proportion of predicted positive pixels that are truly positive, emphasizing the reliability of the predictions; it is commonly used to evaluate a model’s control over false alarms, as defined in Equation (13).


(13) $${Precision}_{k}=\frac{TP_{k}}{TP_{k}+FP_{k}}$$

F1 is the harmonic mean of Precision and Recall, providing a single measure of the trade-off between false alarms and missed detections; in image segmentation, it is equivalent to the Dice coefficient.


(14) $$F1_{k}=\frac{2TP_{k}}{2TP_{k}+FP_{k}+FN_{k}}$$

In addition, to assess model efficiency and deployment friendliness, the number of parameters (Params) and floating-point operations (GFLOPs) are reported. Params reflect the storage cost and structural complexity of the model and are defined as the total number of learnable parameters, as shown in Equation (15):

(15) $$Params=\sum_{l=1}^{L}N_{l}$$

where L denotes the number of network layers and $N_{l}$ represents the number of learnable parameters in the l-th layer. In general, smaller Params indicate lower memory/storage requirements and better suitability for edge deployment.

GFLOPs measure the theoretical computational cost of a single forward pass (in billions of floating-point operations), as defined in Equation (16):


(16) $$GFLOPs=\frac{FLOPs}{10^{9}}$$

Here, FLOPs denote the total floating-point operations required for one forward propagation under a specified input resolution. Lower GFLOPs typically indicate faster inference and lower energy consumption, which is beneficial for real-time or low-compute scenarios.

### 3.3. Results and Analysis

To validate the performance advantages of the proposed model, six representative baselines—TransUNet [29], DeepLabV3 [30], DeepLabV3+ [30], Res-UNet++ [31], U-Net [32], and U-Net++ [33]—were selected for comparison on both the public Crack500 dataset and our proprietary dataset. The results of these experiments, together with those of the proposed DCP-TransUNet, are reported in Table 1.

As shown by the comparative experiments in Table 2, the proposed model demonstrates strong performance on both the Crack500 dataset and our private dataset. In Crack500, because the imaging distance is relatively close, cracks occupy a larger proportion in each image. In contrast, the private dataset was collected by an inspection vehicle. For driving safety, the camera was installed at a higher angle, resulting in a smaller crack proportion in every single frame. Meanwhile, there are many subtle hairline cracks under complex backgrounds, and these factors jointly increase the segmentation difficulty. On the private dataset, an mIoU of 68.83% and an F1 score of 77.57% were achieved; on Crack500, an mIoU of 79.12% and an F1 score of 87.06% were achieved. In both cases, the results exceeded those of the compared networks.

On the private dataset, the task is more challenging due to the smaller crack proportion, more hairline cracks, and complex interference such as shadows, non-uniform illumination, and snow. Compared with the baseline TransUNet, Ours improves mIoU by 3.90% and F1 by 4.30%, while Recall is almost the same and Precision decreases only slightly. This indicates that the gain of our method does not come from more aggressive detections, but more from improvements in structural continuity and boundary alignment, thus significantly improving mIoU and F1. In contrast, DeepLabV3 shows slightly higher Recall but lower Precision, reflecting the typical problem of more detections but more false positives; while TransUNet shows higher Precision but lower mIoU/F1, indicating that its predictions are more conservative and are more likely to miss or break in thin cracks and low-contrast regions. The added CBAM/ECA/SE-UNet shows some improvements compared with the basic U-Net, and SE-UNet achieves the best performance in this series on the private dataset, but it is still lower than Ours overall, suggesting that relying only on lightweight attention enhancement tends to improve feature selectivity and denoising, and still has limitations in maintaining the continuity of slender cracks.

On Crack500, segmentation is generally less challenging because images are captured at a shorter distance and cracks occupy a larger fraction of each frame. Under this setting, the proposed method again achieved the best mIoU and F1. Relative to TransUNet, a more pronounced improvement in Recall was observed, while Precision changed only marginally. This result indicates that missed detections of fine cracks and fragmented crack segments can be substantially reduced with only a limited increase in false positives, thereby improving structural completeness and the region-overlap quality of the predicted crack masks. In comparison, DeepLabV3 attained the highest Recall on Crack500 but suffered from noticeably lower Precision, suggesting a greater tendency to introduce background false positives. Conversely, U-Net and SE-UNet achieved higher Precision but comparatively lower Recall, which is consistent with increased omission of hairline cracks. Overall, a more favorable Precision–Recall trade-off was achieved by the proposed method, leading to more consistent gains in both F1 and mIoU.

To further illustrate the comparative results, six test cases were randomly selected for qualitative visualization: panels (a–c) are drawn from Crack500, and panels (d–f) from the proprietary dataset. The first two columns display the original image and the corresponding ground-truth mask. Columns three through eight present the predicted masks produced by the baseline models used in the comparison.

**Figure 6:**
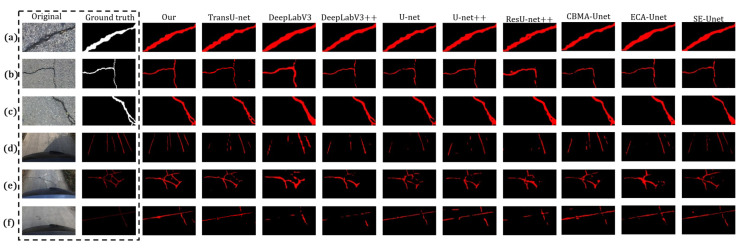
Qualitative comparison of segmentation results on Crack500 (**a**–**c**) and the proprietary dataset (**d**–**f**). Columns 1–2 show the original image and ground-truth mask; columns 3–12 present predictions from the baseline models.

As shown in Figure 6, TransUNet produces clean boundaries and fewer false positives, but it is conservative for low-contrast or extremely narrow cracks, which makes missing details more likely. DeepLabv3 and DeepLabv3+ make fragments easier to connect into an integral whole, but the masks are often thicker and are accompanied by some missed detections and false detections. U-Net and U-Net++ yield tidy outputs but show more obvious missed detections; slender ends tend to become thinner or break. ResUNet++ exhibits partial missed detections and slight outward expansion in regions with complex textures, and the boundaries are not sharp enough. Compared with the basic U-Net, CBAM-UNet, ECA-UNet, and SE-UNet show overall improvements in background denoising and boundary regularity. Their masks are usually cleaner and have a certain ability to repair some broken fragments; however, local discontinuities, missing thin branches, or overly thick predictions can still occur at structural intersections and for hairline cracks. This indicates that enhancement relying only on local attention remains limited in handling long-range connectivity and occlusion-induced breaks of cracks. In contrast, the proposed method (Ours) produces more coherent crack masks overall, with fewer breakpoints in slender branches and clearer boundaries, and less false painting in complex illumination regions such as shadows and specular reflections. It can suppress background interference while maintaining connectivity, further confirming the excellent generalization ability of the proposed model across different datasets.

**Figure 7:**
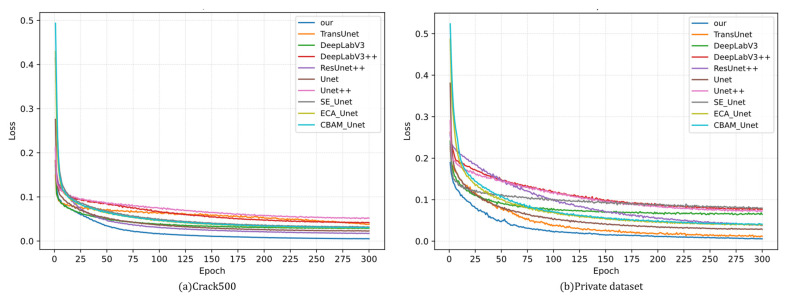
Loss comparison chart.

As shown in Figure 7, the training losses of different models were compared on both Crack500 and the private dataset. On both datasets, a rapid loss decrease was observed during the early training stage, indicating that an initial fit could be achieved effectively by all methods. However, relative to the baseline and other competing approaches, faster convergence was achieved by the proposed method, and a lower asymptotic training loss was consistently maintained throughout the entire optimization process.

On Crack500, the loss of the proposed method dropped below all baselines after the first few epochs and remained lower thereafter, eventually stabilizing at a reduced loss level, whereas most competing methods reached a higher-loss plateau earlier in the mid-to-late training stages. A similar trend was observed on the private dataset: a steeper loss descent and smaller mid-to-late-stage oscillations were exhibited by the proposed method. These observations suggest that slender, discontinuity-prone crack patterns could be learned more effectively while background interference was better suppressed during training, which is consistent with the improvements in mIoU and F1 reported above.

### 3.4. Ablation Experiment

To assess the contribution of each component, an ablation study was conducted on the Crack500 dataset under identical hyperparameter settings. Models were trained with single modules (CLMA, PPA, or DSE), pairwise combinations (PPA+DSE, CLMA+PPA, and CLMA+DSE), and the full configuration integrating all improvements (Ours). Performance was evaluated using mIoU, Recall, F1, and Precision.

**Figure 8:**
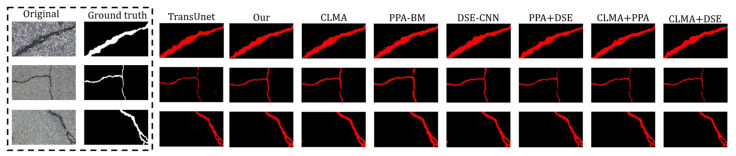
Qualitative ablation results. Columns 1–2 show the original image and ground truth, and columns 3–8 show predictions from the ablation variants.

As summarized in Table 2, the proposed modules consistently improve network performance. Within the TransUNet backbone, incorporating CLMA, PPA-BM, and DSE-CNN yields gains in mIoU, Precision, Recall, and F1-score, indicating enhanced accuracy and robustness for crack detection. Among the single additions, CLMA produces the most pronounced improvement by strengthening long-range continuity of slender cracks and facilitating cross-occlusion recovery. Without CLMA (i.e., PPA+DSE+TransUNet), performance still exceeds the baseline, with mIoU +1.04%, Precision +1.82%, Recall +0.14%, and F1-score +0.84%. Excluding PPA (i.e., CLMA+DSE+TransUNet) likewise improves region overlap and boundary adherence relative to TransUNet, with mIoU +1.12%, Precision +1.62%, Recall +0.45%, and F1-score +0.95%. Removing DSE (i.e., CLMA+PPA+TransUNet) still yields mIoU +1.70%, Precision +0.89%, Recall +1.72%, and F1-score +1.37% over the baseline, reflecting better suppression of irrelevant background and enhancement of discriminative features. When all three modules are integrated (Ours), the network attains the highest overall performance (mIoU = 79.12%, Recall = 87.96%, F1 = 87.06%), with Precision (86.21%) close to the baseline, demonstrating more accurate and robust crack segmentation under challenging conditions such as tree shadows and nonuniform illumination, see Figure 8.

To more clearly elucidate the differences and contributions of each module, we further provide a visual comparison of different module combinations on the crack segmentation task, as shown in Figure 7. The DSE-CNN module is primarily designed to enhance feature selectivity and suppress noise, with its main contribution reflected in reducing false positives. Specifically, DSE-CNN employs dual-channel recalibration to amplify informative semantic channels while attenuating channels dominated by fine-grained textures and noise. As a result, the network is encouraged to preserve genuine crack responses and to suppress spurious activations under complex textured backgrounds. In Figure 7, this effect is visually evidenced by fewer scattered responses in background regions and a more complete and clearer crack mask.

The PPA-Bottleneck module, in contrast, is mainly intended to strengthen token–pixel alignment and boundary adherence, thereby improving contour quality and region overlap. Positioned at the encoder–decoder bridging stage, this module enhances the consistency between token representations and pixel-domain details, which enables crack boundaries to better conform to the underlying structures and yields more regular crack shapes. Consequently, boundary leakage is effectively mitigated and contour consistency is improved, leading to more stable gains in region overlap. Correspondingly, the PPA-based results in Figure 7 exhibit superior stability in crack width and smoother boundary delineation.

The CLMA module is more oriented toward preserving the long-range continuity and topology of thin and elongated cracks, and it plays a critical role in the overall performance improvement. Unlike the DSE and PPA modules, which mainly focus on local details, CLMA directly targets the structural issues that are most sensitive in crack segmentation. Thin cracks are prone to fragmentation under occlusion, low contrast, and local perturbations; such breakages can severely damage connectivity and consequently lead to a pronounced drop in mIoU. Directional aggregation is performed via cross-stripe attention within horizontal and vertical stripe windows, such that local features are propagated preferentially along the principal crack direction. Meanwhile, global memory cross-attention leverages a small set of stable memory tokens to provide a compact global context, which suppresses strong local disturbances (e.g., shadows, specular highlights, and background textures) while aligning dispersed fragments across windows. This design more effectively bridges occlusion-induced structural discontinuities and reduces spurious connections.

## 4. Conclusions

To address the challenge of segmenting cracks that are thin, elongated, and prone to fragmentation under complex interference, such as shadows, specular highlights, and local occlusions, DCP-TransUNet is proposed. A novel encoder is designed to improve channel selectivity by integrating a CNN with Dual Squeeze–Excitation. The encoder is followed by a CLMA-Transformer block, where directional dependencies and global context are jointly modeled through horizontal–vertical cross-stripe attention and a small set of memory tokens. At the bridging stage, a PPA-bm module is introduced to align token-level and pixel-level representations, strengthen boundary adherence in discriminative features, and enhance the continuity of fragmented crack structures.

Experimental results demonstrate consistent improvements over strong baselines. On Crack500, an mIoU of 79.12% is achieved, along with a Recall of 87.96%, an F1-score of 87.06%, and a Precision of 86.21%. On the proprietary dataset, an mIoU of 68.83% is obtained, together with a Recall of 74.42%, an F1-score of 77.57%, and a Precision of 81.67%. These results indicate that robust and accurate crack segmentation can be delivered by the proposed model, thereby providing a high-performance solution for roadway safety maintenance. In addition to improving the accuracy and efficiency of pavement-distress detection, overall maintenance costs may be reduced.

Shortcomings and future research directions:

1. Although consistent improvements were obtained by DCP-TransUNet on Crack500 and a private dataset, performance fluctuations may still be observed when the data acquisition conditions deviate from the training distribution. Such domain shifts may arise from differences in pavement materials and textures, variations in camera height and viewing angle, seasonal and illumination changes, and heterogeneous imaging characteristics across sensors. Future work will therefore focus on cross-domain robust learning, with emphasis placed on self-supervised pretraining using large-scale unlabeled road images and on domain adaptation techniques, so that the sensitivity of the model to scene variations can be reduced.
2. While the superior capability of DCP-TransUNet was validated experimentally, additional computational cost and GPU memory consumption are introduced by the proposed method, which may constrain real-time deployment on edge devices. In future work, lightweight architectural designs and inference acceleration strategies will be investigated to reduce model complexity and improve real-time performance while maintaining accuracy as much as possible.
3. In addition, a staged validation on real roads will be conducted to systematically assess stability, real-time performance, and practical utility under operational conditions. In the first stage, on-device deployment on an embedded computing platform and testing in a controlled road environment will be performed to verify end-to-end inference speed, resource utilization, and baseline robustness. In the second stage, the model will be further integrated into a practical road inspection system or an in-vehicle data acquisition platform, followed by long-term testing and comparative evaluation under real traffic and multiple interference conditions. Particular attention will be paid to the overall performance in terms of crack detection completeness, false-positive control, operational safety, and the effectiveness of maintenance decision support.

## Figures and Tables

**Table 1:** Experimental Results.

Dataset	Model	MIoU	Recall	F1	Pre	Params(M)	GFLOPs
Private dataset	our	68.83%	74.42%	77.57%	81.67%	174.662	69.656
	TransUNet	64.93%	74.27%	73.27%	82.80%	105.276	50.725
	DeepLabV3	65.69%	75.34%	73.79%	73.21%	39.634	62.82
	DeepLabV3+	65.13%	70.30%	72.95%	78.03%	39.757	17.684
	Res-UNet++	63.42%	70.10%	68.85%	73.21%	14.483	108.629
	U-Net	64.96%	68.24%	73.31%	82.45%	17.263	61.457
	U-Net++	65.18%	71.21%	72.79%	76.63%	26.905	57.648
	ECA-UNet	65.11%	70.26%	72.91%	78.03%	31.385	85.589
	CBMA-UNet	65.55%	71.27%	73.71%	77.62%	31.604	85.665
	SE-UNet	66.80%	70.92%	75.39%	82.39%	31.606	85.590
Crack500	our	79.12%	87.96%	87.06%	86.21%	174.662	69.656
	TransUNet	76.4%	82.62%	84.88%	87.51%	105.276	50.725
	DeepLabV3	77.29%	90.79%	85.67%	81.78%	39.634	62.82
	DeepLabV3+	77.16%	84.16%	85.51%	86.98%	39.757	17.684
	Res-UNet	76.37%	87.03%	84.90%	83.02%	14.483	108.629
	U-Net	76.87%	82.68%	85.27%	88.34%	17.263	61.457
	U-Net++	76.92%	84.43%	85.32%	86.25%	26.905	57.648
	ECA-UNet	77.43%	84.22%	85.73%	87.38%	31.385	85.589
	CBMA-UNet	77.37%	84.16%	85.67%	87.34%	31.604	85.665
	SE-UNet	76.94%	82.88%	85.32%	88.20%	31.606	85.590

**Table 2:** Ablation Experimental Results.

Model	MIoU	Recall	F1	Pre
transunet	76.4%	82.62%	84.88%	87.51%
CLMA	76.63%	85.72%	85.10%	84.50%
PPA-BM	76.76%	83.85%	85.19%	86.64%
DSE-CNN	76.66%	82.63%	85.09%	88.00%
PPA+DSE	77.44%	82.76%	85.72%	89.33%
CLMA+PPA	78.10%	84.34%	86.25%	88.40%
CLMA+DSE	77.52%	83.07%	85.83%	89.13%
our	79.12%	87.96%	87.06%	86.21%

## Data Availability

The original contributions presented in the study are included in the article; further inquiries can be directed to the corresponding author.
